# Custom Ocular Prosthesis With Intraoral Obturator for Orbital and Intraoral Defects Resulting From Mucormycosis: A Technical Case Report

**DOI:** 10.7759/cureus.73579

**Published:** 2024-11-13

**Authors:** Anshul Chugh, Mariko Hattori, Yuka I Sumita, Noriyuki Wakabayashi

**Affiliations:** 1 Prosthodontics, Crown and Bridge, Postgraduate Institute of Dental Sciences, Haryana, IND; 2 Advanced Prosthodontics, Institute of Science Tokyo, Tokyo, JPN; 3 Partial and Complete Denture, The Nippon Dental University, School of Life Dentistry at Tokyo, Tokyo, JPN

**Keywords:** custom made jigs, eye prosthesis, iris orientation, maxillofacial prosthesis, mucormycosis, obturator, two-piece prosthesis

## Abstract

Orbital defects can result from a myriad of underlying diseases and injuries. Treatment of malignant neoplasms and maxillofacial trauma are common reasons for orbital exenteration. Recently, a growing number of cases of orbital defects have been reported due to mucormycosis in patients with COVID-19. In this report, we describe a technique to achieve ideal iris orientation in the fabrication of a silicone orbital prosthesis using custom-made eyeglasses as a jig. This method allows for easy orientation and reorientation, enabling precise iris alignment and enhancing the aesthetic outcome.

## Introduction

Orbital defects can arise from various underlying diseases and injuries. Orbital exenteration is often necessary for treating malignant tumors and maxillofacial trauma [1].

Recently, numerous cases of orbital mucormycosis as a complication of COVID-19 have been reported. Mucormycosis, also known as phycomycosis, is a fungal infection that typically affects the upper respiratory tract and can become severe in immunocompromised individuals [1,2]. An upper respiratory tract infection can spread to the paranasal sinuses and may then easily spread to the orbit and brain [3-5]. True orbital mucormycosis is a rare condition typically associated with an initial infection in the sinonasal region as a result of invasion of the orbital wall from the paranasal sinuses. An increase in these cases has been observed during the COVID-19 pandemic [6,7]. Mucormycosis is divided into six types based on anatomic location: rhinocerebral, pulmonary, cutaneous, gastrointestinal, disseminated, and uncommon presentations (endocarditis, osteomyelitis, peritonitis, and pyelonephritis) [8].

Although amphotericin B has proven efficacy in the treatment of mucormycosis, the relentless progression of mucormycosis requires extensive surgical measures, such as exenteration to remove the orbital contents, resulting in complex maxillofacial defects and challenges related to morphology, functionality, and adverse psychosocial effects. In the rehabilitation phase, addressing these issues involves either surgical or prosthetic approaches [9]. While surgical reconstruction is considered the superior rehabilitation technique, prosthetic intervention offers advantages such as simplicity, reduced invasiveness, and the ability to monitor for disease recurrence, making it particularly suitable for frail and elderly patients [10,11]. However, fabricating an orbital prosthesis, whether alone or in combination with an obturator, can pose significant challenges.

In this report, we discuss the fabrication of an orbital prosthesis for a patient who underwent orbital exenteration, with a focus on both functional and aesthetic improvements. The objective of prosthetic rehabilitation was to support the biological health of the underlying postsurgical tissue bed, ensure the longevity of the prosthesis, and achieve satisfactory aesthetic outcomes.

The primary focus of our report is on the use of custom-made eyeglasses and a custom sprue as jigs for easier orientation and reorientation. This technique enabled precise iris alignment, enhanced aesthetic outcomes, and improved the visual appeal of the prosthesis.

The prosthesis was fabricated by combining heat-polymerized polymethyl methacrylate as a substructure and room-temperature vulcanizing silicone prosthetic material with the help of customized jigs. In this report, we describe the innovative prosthetic design to enhance both retention and aesthetic outcomes, as well as our efforts to refine the prosthesis, aiming for improved functionality and a more visually appealing result. The study was conducted in accordance with the Declaration of Helsinki, and written informed consent was obtained from the patient.

## Case presentation

The patient was a 50-year-old man who had orbital and maxillary defects due to mucormycosis as a complication of a COVID-19 infection. He was referred for prosthetic rehabilitation following a partial maxillectomy and exenteration on the left side. Due to the connection between the oral cavity, nasal cavity, and orbit, he experienced difficulties with speech, mastication, and swallowing and also had concerns about his appearance. The fabrication of intraoral and extraoral prostheses was planned.

**Figure 1:**
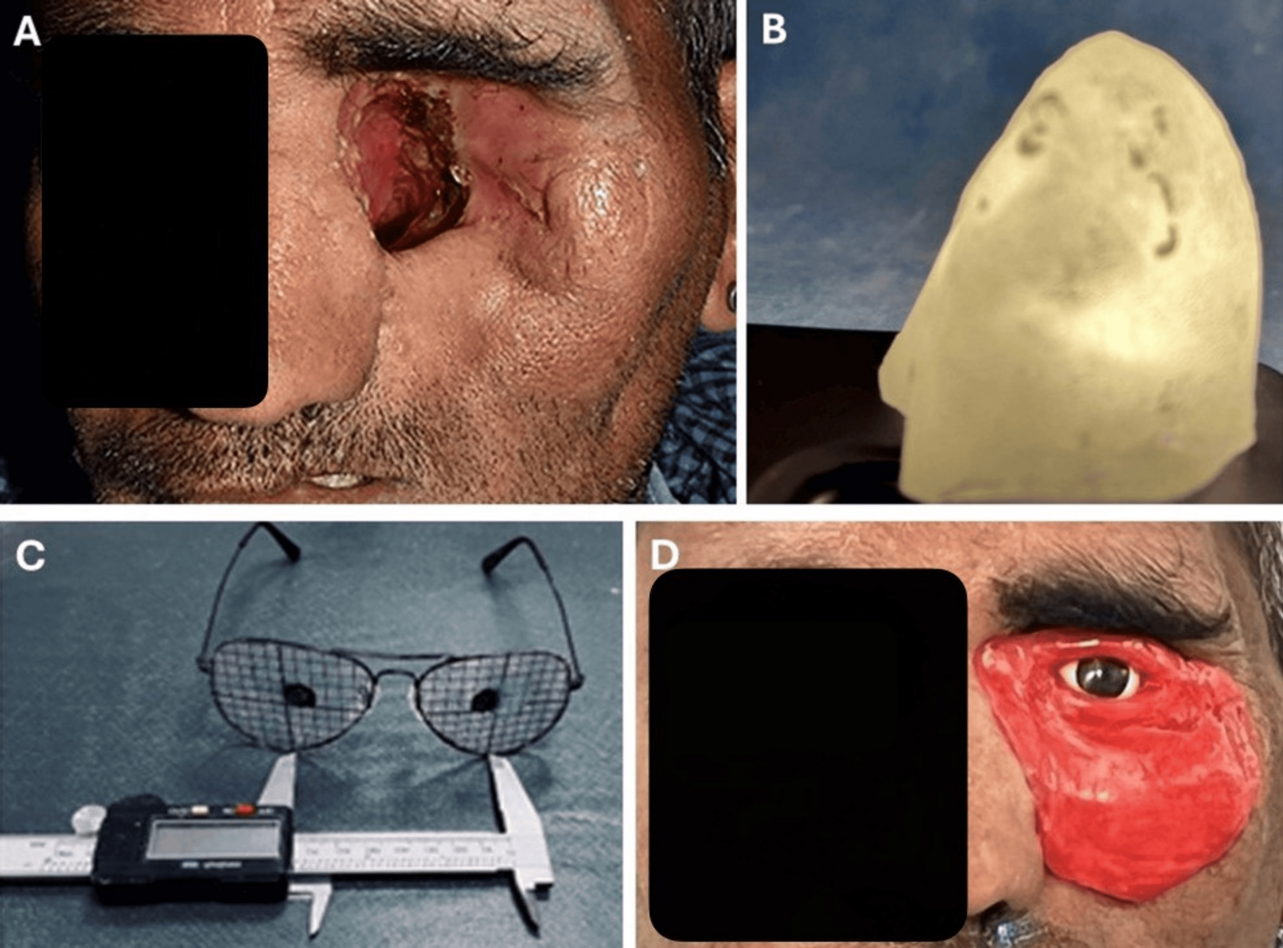
The fabrication of prostheses A: orbital defect; B: light passing through hollow substructure; C: custom-made eyeglasses for iris orientation; D: wax try-in with the oriented iris

The orbital and oral defects were examined (Figure 1A). The diagnostic process for the orbital defect was initiated by making an impression with impression compound (Impression Tray Compound, GC Corp., Tokyo, Japan). A stone model was constructed to assess the defect, and a custom tray was fabricated. The process of creating retention holes in the custom tray was done. Adhesive (Universal tray adhesive, GC Corp., Tokyo, Japan) was applied to the tray, and then the definitive impression was made with regular viscosity hydrophilic polyvinyl siloxane (Examix Fine Regular, GC Corp., Tokyo, Japan). The tray was inserted into the defect to ensure proper positioning and removal. The definitive impression was made into stone molds using Type IV dental stone (New Fuji Rock, GC Corp., Tokyo, Japan). The impression was removed, and the molds were coated with a separating medium (Acro Sep, GC Corp., Tokyo, Japan). The fabrication of the substructure using heat-activated polymerizing acrylic resin (PalaPress Vario, Kulzer GmbH, Hanau, Germany) was completed, following the manufacturer’s instructions. A hollow substructure was created and checked by passing light through it (Figure 1B). The prefabricated eye shell was selected by referencing the patient’s normal eye. The eye shell was adjusted using custom-made eyeglasses as a jig. Grids were created with a marker, and the iris was positioned in relation to the normal eye (Figure 1C). A wax-up was performed with the iris in place, and a wax try-in was conducted with the patient using the custom-made eyeglass jig for proper iris positioning. The normal eye was simulated on the opposite side (Figure 1D). The wax-up was articulated in a dental flask using a two-pour technique. A custom-made sprue was fabricated using auto-polymerizing resin and fixed to the iris to maintain its position after dewaxing. The iris was placed in the same orientation within the maxillofacial silicone prosthesis. Room-temperature silicone elastomer was mixed in a 10:1 ratio (VST-50-F, Factor II), and oil-based colors (Camel oil colors, Kokuyo Camlin, Mumbai, India) were used for intrinsic coloration, matching the silicone to the patient’s skin color. In the acrylic mold, 2 mm retention holes were made with a bur, cleaned with acetone, and allowed to dry. A thin coating of primer (A-330-5, Factor II) was applied. The mold was packed with VST-50-F mixed with the skin tone color and polymerized for 24 hours according to the manufacturer's instructions. Excess material was removed using silicone trimming burs and wheels. The dismantled iris was placed back in the same position with the help of the custom sprue, which was then cut. The orbital prosthesis was polished using dental polishing burs (Big Point, KDF Denken-High Dental Co. Ltd., Kyoto, Japan) and waterproof abrasive paper (Fuji Star, Sankyo-Rikagaku Co. Ltd., Okegawa, Japan).

**Figure 2:**
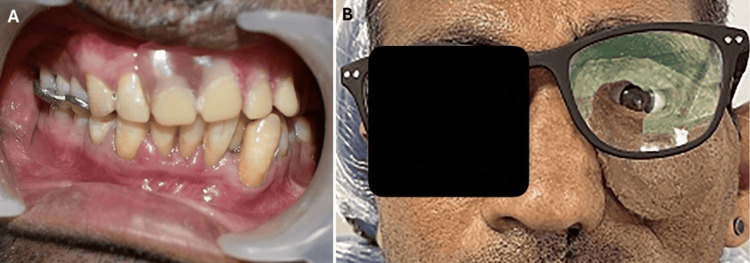
Prostheses in place A: obturator for the intraoral defect; B: view of the delivered orbital prosthesis

The finished prosthesis was delivered with maintenance instructions, showing the intraoral view of the inserted obturator (Figure 2A). In cases with both oral and orbital defects, an intraoral maxillary obturator was initially created with an orbital prosthesis, connecting both with intraoral magnets. Later, if the patient already had an obturator, only an orbital prosthesis was made, with intraoral magnets attached to the prosthesis. The fit of the prosthesis in the defect was evaluated, and any discomfort was corrected. The use of eyeglasses was recommended for cases where intraoral retention was achieved with magnets and the fit was good, eliminating the need for adhesive (Figure 2B). After follow-up, he was satisfied with the function and appearance, as he regained a social life close to normal.

## Discussion

Conventional orbital prostheses can be created using various techniques. However, these techniques often do not address the orientation or replacement of the iris. The prosthesis, while not functional, serves as an effective aesthetic replacement for patients, significantly enhancing their self-confidence and alleviating social embarrassment. This technique outlines the fabrication process of prostheses using materials that are easily accessible and routinely employed by maxillofacial prosthetic specialists. The procedure is designed to ensure a proper fit for the artificial eye while also achieving a natural aesthetic outcome [10]. As a result, these prostheses often have issues with fit, size, and alignment with the normal eye. The custom-made orbital prosthesis described in this report was fabricated using a custom-made eyeglass jig and sprue iris retainer. This approach reduced issues related to the alignment of the prosthetic iris with the normal eye and facilitated the proper positioning of the iris in the final stages of prosthesis fabrication. Commercially available skin-matching colors are expensive and not easily accessible. Since silicone elastomer is an oil-based material, oil-based colors have been tested and provided promising, affordable results. With this custom-made orbital prosthesis, the orientation of the iris was aligned with the patient’s normal eye. Additionally, the fit was improved, and the use of intraoral magnets or eyeglasses with adhesive was comfortable for the patient [11]. The application of magnets to improve the retention of maxillofacial prostheses was first introduced by Nadeau, who innovatively utilized magnets to connect intraoral and extraoral prosthetic components [12]. Compared with conventional types of prostheses where procedures are done arbitrarily based on the operator’s visual skill, the custom-made prostheses had less extra-oral coverage while utilizing intraoral magnets for retention and ensuring proper iris orientation. This lightweight and narrower prosthesis enhanced the patient’s comfort, confidence, and appearance compared with oversized prostheses made by conventional techniques. The limitations of silicone prostheses include issues such as delamination, material degradation, and compromised marginal integrity. However, these challenges can be effectively addressed through meticulous treatment planning and comprehensive patient education. By implementing a structured approach to the design and fitting of the prosthesis, clinicians can enhance its performance and longevity [13].

## Conclusions

This case report demonstrates a novel technique for achieving ideal iris orientation in the fabrication of a silicone orbital prosthesis using custom-made eyeglasses as a jig. The technique facilitates easy orientation and reorientation, ensuring precise iris alignment significantly enhancing the aesthetic outcome, and increasing patient satisfaction. This approach makes a valuable contribution to the field of prosthetic rehabilitation, offering a reliable and economical method for enhancing both the functionality and aesthetics of orbital prostheses.
